# Muscle, Neuromuscular, and Cardiac Damage in Trail Running: A Systematic Review

**DOI:** 10.3390/muscles5010009

**Published:** 2026-01-29

**Authors:** Isabel García-Valiente, Francisco Pradas, Miguel Ángel Ortega-Zayas, Carlos Castellar-Otín, Alejandro García-Giménez, Miguel Lecina

**Affiliations:** 1 ENFYRED Research Group, Faculty of Health and Sports Sciences, University of Zaragoza, 22002 Huesca, Spain; 797752@unizar.es (I.G.-V.); maortega@unizar.es (M.Á.O.-Z.); mlecina@unizar.es (M.L.); 2 Faculty of Social Sciences and Humanities, University of Zaragoza, 44003 Teruel, Spain; 3 Faculty of Health Sciences, San Jorge University, 50830 Villanueva de Gallego, Spain

**Keywords:** endurance, natural environment, muscle damage, biomarkers, contractile efficiency

## Abstract

**Background**: Trail running (TR) is an endurance discipline characterized by prolonged exercise, irregular terrain, and marked elevation changes, which increase eccentric muscular load and may induce muscular, neuromuscular, and cardiac damage. **Objective**: This study aimed to systematically review the evidence on muscular, neuromuscular, and cardiac damage associated with TR participation. **Methods**: This systematic review followed PRISMA 2020 guidelines and was registered in PROSPERO (CRD420251135043). Five databases (PubMed, Web of Science, Scopus, SportDiscus, and ScienceDirect) were searched up to 31 August 2025. Observational, longitudinal, prospective, and case studies involving healthy adolescent or adult trail runners were included. Outcomes comprised muscle damage biomarkers (e.g., creatine kinase, alanine aminotransferase), neuromuscular function (e.g., squat jump performance, maximal voluntary isometric contraction), and cardiac biomarkers (e.g., CK-MB, cardiac troponins, NT-proBNP). Methodological quality was assessed using the National Heart, Lung, and Blood Institute Study Quality Assessment Tool. Results were synthesized qualitatively. **Results**: Fifteen studies met the inclusion criteria, including a total of 247 participants. Post-race analyses consistently showed marked increases in muscle damage biomarkers and significant reductions in neuromuscular performance. Transient elevations in cardiac biomarkers were also observed, suggesting acute but reversible cardiac stress following TR events. **Limitations**: Evidence was limited by methodological heterogeneity, small sample sizes, and underrepresentation of female athletes. **Conclusions**: It was found that trail running induces substantial acute muscular, neuromuscular, and cardiac stress, particularly in events with high eccentric loading. Monitoring biochemical and neuromuscular markers may support training load optimization, recovery strategies, and injury prevention.

## 1. Introduction

Trail running (TR) is a discipline that combines running with the challenge of traversing natural mountainous environments with varying levels of technical difficulty. Unlike running on smooth surfaces, this modality exposes athletes to substantial elevation changes, irregular terrain, variable weather conditions, and natural obstacles throughout the course [1]. This constantly changing and unpredictable environment requires greater physical and mental effort, not only due to the need for continuous adaptation but also because of the increased risk of accidents and/or injuries [2]. In recent years, this discipline has experienced significant growth at both recreational and competitive levels, reflected in the increasing number of official events and participants [3]. Due to its characteristics, TR attracts a wide population range—from young individuals to older adults—and is practiced by both men and women [4]. However, this expansion has also generated uncertainty, as the risk factors associated with TR vary considerably depending on age, sex, previous experience, and training level [5].

Compared with road running, TR requires athletes to cope with uneven terrain, elevation gain and loss, and fluctuating environmental conditions, all of which greatly increase the likelihood of sustaining injuries. Injury incidence is higher during competitions than training, particularly in ultra-distance events [6,7]. The type and distribution of injuries vary according to the nature of the event; multistage competitions show a predominance of knee injuries, whereas continuous long-distance races present higher rates of foot and ankle injuries [8,9]. In general, the most frequent injuries in TR affect the lower limbs and include ankle sprains, Achilles and patellar tendinopathies, various types of muscular overload, and even stress fractures [10,11].

One of the most relevant aspects of injury analysis in TR is muscle damage induced during performance—particularly during the eccentric phase of muscle contraction that occurs on downhill sections, where mechanical stress on muscle fibers is substantial [12,13]. During eccentric contractions, the muscle lengthens while producing tension, which can lead to sarcomere disruption, cell membrane damage, and the release of muscle damage biomarkers, such as creatine kinase (CK), lactate dehydrogenase (LDH), alanine aminotransferase (ALT), and myoglobin (MB). These responses reflect structural microdamage and inflammation [14]. This downhill-related eccentric stress compromises not only muscle tissue integrity but also neuromuscular function, altering motor coordination, reducing maximal voluntary force, and increasing both peripheral and central fatigue [15].

Neuromuscular fatigue—defined as a transient decline in the neuromuscular system’s ability to generate force—is closely related to muscle damage, with both phenomena influencing one another [16]. Damaged muscle exhibits reduced contractile efficiency, potentially increasing mechanical load on other structures and contributing to compensatory patterns that elevate injury risk [17]. Reduced motor control may also lead to technical errors, further increasing the likelihood of musculoskeletal injuries [16]. In TR, neuromuscular function can be profoundly affected due to irregular terrain, frequent pace changes, and prolonged uphill and downhill segments [18].

Similarly to the muscle damage biomarkers described above (CK and LDH), several studies have associated muscular fatigue with substantial reductions in lower-limb strength [19,20]. These alterations—particularly in ultra-trail events—can even progress to severe conditions such as rhabdomyolysis or myopathies [21]. In fact, the literature reports extremely pronounced muscular alterations in TR, in some cases exceeding those documented in high-intensity sports such as CrossFit [22], where cardiac muscle has also shown acute stress responses [23,24].

Cardiac stress has been investigated in TR, especially in ultra-distance races [25], through specific biomarkers and diagnostic imaging techniques, including echocardiography, magnetic resonance imaging and computed tomography [26]. These methods provide parameters such as the left ventricular ejection fraction (LVEF), a key marker of cardiac contractile function, which is typically altered in pathologies such as heart failure or myocardial infarction [27,28]. LVEF represents the percentage of blood ejected from the left ventricle with each heartbeat relative to its end-diastolic volume [29] with normal values ranging from 55–70% [30]. While moderate aerobic exercise positively affects LVEF compared with sedentary individuals [31], elite athletes may present similar or slightly reduced values due to chronic physiological adaptations [32,33]. Another widely studied parameter is heart rate variability (HRV), which reflects parasympathetic regulation and has been linked to cardiac stress and autonomic imbalance during high-intensity efforts, though its prognostic value in ultra-endurance running remains debated [34]. Over the past years, advances in clinical cardiology have led to the development of new biomarkers to help diagnose cardiac stress in athletes, including cardiac troponin (cTn), high-sensitivity troponin (hs-cTn), creatine kinase MB isoenzyme (CK-MB), and N-terminal pro–B-type natriuretic peptide (NT-proBNP) [35]. The increase in these biomarkers during TR suggests that athletes are exposed to specific systemic risks that produce transient cardiac stress without necessarily implying permanent myocardial injury [36,37,38]. However, these risks may be exacerbated under conditions such as dehydration and/or fatigue [39]. The literature also highlights multiple injury risk factors in TR, including experience level, terrain type, footwear selection and individual characteristics of runners [5]. Although several preventive measures have been proposed—such as strength training, improved downhill technique, and proprioceptive work—the evidence supporting their effectiveness remains limited, especially for women and non-professional athletes [40].

Taken together, trail and ultra-endurance running are characterized by marked but largely transient alterations in blood biomarkers that reflect the combined muscular, neuromuscular, cardiac, inflammatory, and metabolic stress imposed by these events. Across studies, elevations in muscle damage markers have been consistently associated with substantial, short-term reductions in lower-limb strength and neuromuscular performance, with recovery timelines ranging from several days to more than one week after prolonged or multi-stage competitions [37,41]. In parallel, ultra-trail races elicit acute increases in cardiac biomarkers, sometimes exceeding clinical reference values, which generally reflect transient myocardial stress without evidence of persistent structural damage in healthy runners [42,43,44]. Overall, the magnitude and temporal profile of these biomarker responses appear closely linked to acute performance impairment and recovery demands, with potential implications for subsequent training tolerance and adaptation.

Given this background, this review aimed to synthesize evidence on muscular, neuromuscular, and cardiac damage responses in healthy trail runners following competition or simulated trail running events.

## 2. Materials and Methods

### 2.1. Criteria for Study Search and Selection

This study consisted of a systematic review of the available scientific evidence related to muscle damage in TR. The review followed the Preferred Reporting Items for Systematic Reviews and Meta-Analyses (PRISMA) guidelines [45] and was registered according to the recommendations of the National Institute for Health and Care Research (NIHR) in the International Prospective Register of Systematic Reviews (PROSPERO), under registration code CRD420251135043, accessible at https://www.crd.york.ac.uk/PROSPERO/view/CRD420251135043, accessed on 27 August 2025. No amendments to the review protocol were made after registration.

Articles related to the research topic and published during the last 15 years were selected, with the search cutoff date set for 31 August 2025. A structured and selective search was carried out in the health and sport sciences fields using five databases: PubMed, Web of Science, Scopus, Sport Discus, and ScienceDirect. The search strategy combined medical subject headings (MeSH) and free-text terms associated with trail running (“trail running” OR “mountain running”) AND (injury). The final search equation included the following keywords: (“Trail Running” OR “trail running” OR “ultra-trail” OR “mountain running”) AND (“Muscle Damage” OR “muscle injury” OR “exercise-induced muscle damage”) AND (“Neuromuscular Fatigue” OR “neuromuscular function” OR “neuromuscular fatigue”) OR (“Cardiac Biomarkers” OR “troponin” OR “CK-MB” OR “NT-proBNP” OR “high-sensitivity cardiac troponin I”) AND (“Prevention” OR “performance” OR “recovery”). The full electronic search strategy for PubMed is provided in Supplementary Material Table S1.

No trial registers, preprint servers, or gray literature sources were searched.

### 2.2. Inclusion Criteria

Studies were eligible if they involved healthy adolescent or adult trail runners and assessed muscular, neuromuscular, or cardiac damage using biochemical or functional outcomes. Studies were grouped according to the primary damage domain assessed.

To ensure methodological quality, the following inclusion criteria were established:

1. Case studies, observational studies, longitudinal studies, or prospective designs.
2. Adolescent or adult populations of both sexes (>15 years old) without medical conditions or pathology.
3. Studies published between 2010 and 2025.
4. Studies written in English or Spanish.
5. Publications analyzing muscle damage using blood biomarkers such as creatine kinase (CK), lactate dehydrogenase (LDH), or AST/ALT (Aspartato Aminotransferasa/Alanine Aminotransferase); peripheral neuromuscular fatigue evaluated using dynamic tests of lower-limb elastic–explosive strength (SJ, CMJ, ABA) or maximal voluntary isometric contractions (MVIC); and cardiac damage assessed through biomarkers including Pro-BNP, CK-MB, cTnI, high-sensitivity cardiac troponin I (hs-cTnI), and N-terminal pro–brain natriuretic peptide (NT-proBNP).

For the purposes of this review, trail running was operationally defined to include outdoor trail and mountain races, ultra-trail and multi-stage off-road events, as well as controlled treadmill protocols designed to replicate the eccentric and mechanical demands characteristic of trail running.

### 2.3. Exclusion Criteria

The following exclusion criteria were applied:

1. Studies that did not explicitly refer to trail running;
2. Publications that did not address muscle damage or muscle fatigue, defined as contractile force loss or cardiac damage;
3. Participants reporting any cardiac pathology or previous muscular injury.

### 2.4. Data Extraction

Two authors (I.G.-V. and F.P.) independently screened the articles identified through the selected databases. Titles and abstracts were screened first, followed by full-text assessment. No automation tools were used in the selection process. During the review process, the following information was extracted from each study: publication year; authors; participant characteristics (sample size, age, performance level, sex); type of event (distance, number of stages, positive elevation gain); muscle damage biomarkers (CK and ALT); cardiac biomarkers (CK-MB, Pro-BNP, cTnI, hs-cTnI and NT-proBNP); and neuromuscular outcomes such as reductions in squat jump (SJ) performance and maximal voluntary isometric contraction (MVIC). For each outcome, all reported time points (pre-, post-race, and recovery) were extracted when available. After applying the inclusion and exclusion criteria, two authors (I.G.-V. and F.P.) independently extracted all data using Microsoft Excel^®^ 2024 (Microsoft Corporation, Redmond, WA, USA). No assumptions were made for missing or unclear data, and no data were imputed. Disagreements were resolved through discussion, and when necessary, a third reviewer (M.Á.O.-Z.) participated to reach consensus.

No standardized effect measures or quantitative effect sizes were calculated.

### 2.5. Overall Quality of Included Studies

**Figure 1:**
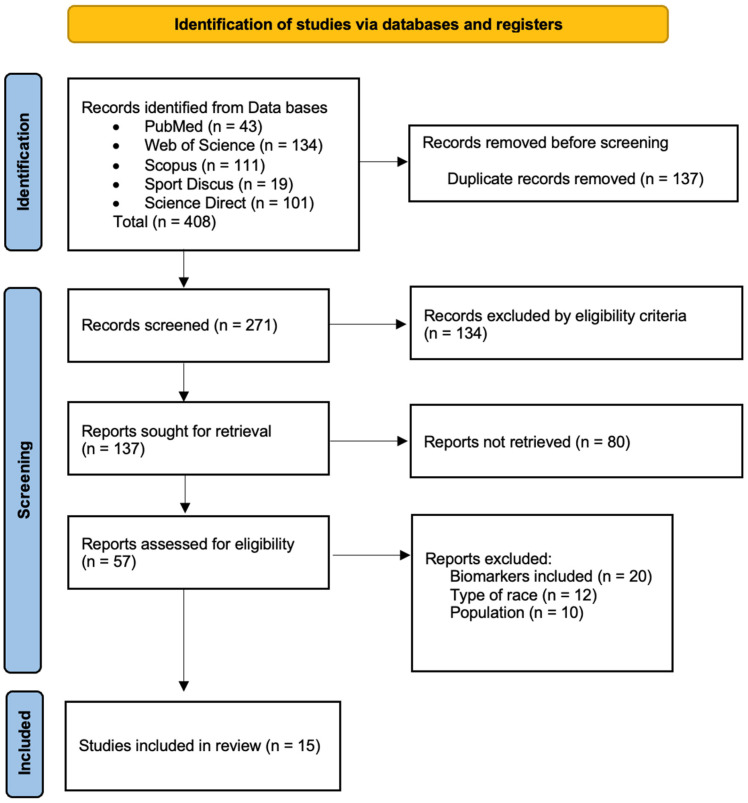
Flow diagram describing the study selection process.

The articles included were screened by two independent reviewers (I.G.-V. and M.L.) based on the predefined inclusion and exclusion criteria. Duplicate articles were removed using Mendeley Desktop^®^ v.2112.0 (Elsevier, Amsterdam, The Netherlands), and titles and abstracts were analyzed. When required, full texts were consulted for additional evaluation. All decisions were approved by both reviewers; disagreements were resolved by consulting a third reviewer (M.Á.O.-Z.). The full analysis process lasted four weeks. A detailed summary of the selection process is shown in Figure 1.

### 2.6. Synthesis Methods

Given the heterogeneity in study designs, outcome measures, and reporting formats, a quantitative synthesis (meta-analysis) was not performed. Instead, a narrative qualitative synthesis was conducted. Studies were grouped according to the primary domain assessed (muscular damage, neuromuscular function, or cardiac damage), and results were summarized descriptively across studies.

### 2.7. Risk of Bias Assessment and Methodological Quality

Most studies included in this review were observational with descriptive designs. Due to their non-experimental nature, tools such as the PEDro scale [46] or the Cochrane Collaboration tool [47], commonly used in clinical trials, could not be applied. Instead, methodological quality was assessed using tools adapted to observational descriptive studies, focusing on qualitative criteria [48].

**Figure 2:**
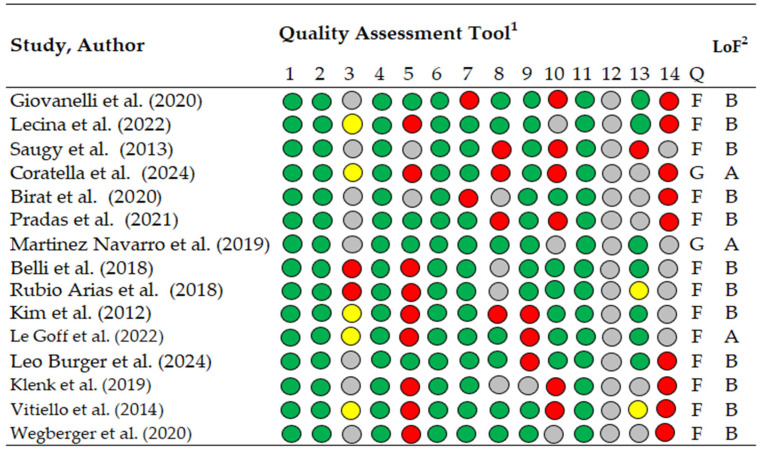
Methodological quality assessment of included studies using the NHLBI Study Quality Assessment Tool for Observational Cohort and Cross-Sectional Studies Green indicates “yes”, red “no”, yellow “not reported”, and gray “cannot determine”. Level of evidence categories were defined as A = good methodological quality and B = fair methodological quality, following previously published criteria, ^1^ Quality assessment tool for Observational Cohort and Cross-Sectional Studies, ^2^ LoF; Level of Evidence, 1–14, check list criteria; Q, quality; G, good; F, fair [14,37,41,43,44,45,49,50,51,52,53,54,55,56,57].

Methodological quality was evaluated using the National Heart, Lung, and Blood Institute (NHLBI) Study Quality Assessment Tool (Figure 2), available at https://www.nhlbi.nih.gov/health-topics/study-quality-assessment-tools, accessed on 9 November 2025. This tool was selected due to the predominance of observational and descriptive study designs. It includes 14 items rated as “yes” (green), “no” (red), “not reported” (yellow), or “cannot determine” (gray). Studies were categorized as good (>11 “yes” items), fair (7–10 “yes” items), or poor (<6 “yes” items). The 14 items were assessed independently by two authors (M.L. and A.G.-G.), and disagreements were resolved through consensus or, when necessary, arbitration by a third reviewer (M.Á.O.-Z.).

### 2.8. Reporting Bias Assessment

Formal assessment of reporting bias was not conducted due to the absence of a quantitative synthesis and the limited number of studies available for each outcome.

### 2.9. Certainty of Evidence

The certainty of evidence was not formally assessed using tools such as GRADE due to heterogeneity in study designs, outcomes, and reporting formats.

## 3. Results

A total of 408 potentially relevant studies were initially identified (Figure 1). After removing duplicates, 137 articles remained. The inclusion and exclusion criteria were then applied through title and abstract screening, resulting in 80 studies selected for full-text evaluation. Of these, 57 were classified according to their main thematic focus. Finally, fifteen studies were included in this review, selected for specifically addressing the effects of TR on muscle damage, neuromuscular function, and/or cardiac stress. Reasons for exclusion of full-text articles are reported in the PRISMA flow diagram (Figure 1).

### 3.1. Studied Population and Type of Event

Table 1 presents the included investigations, detailing the characteristics of the study populations and the events analyzed. A total of 15 studies involving 247 participants, 30 of them females, were included. Regarding age, studies reported mean or median ages, generally ranging between 30 and 50 years, although one study included adolescent participants aged 15–20 years. Only one of the fifteen studies differentiated participants based on performance level, categorizing them as amateur versus high-level runners.

### 3.2. Muscle Damage Markers

Table 2 summarizes the biomarkers analyzed across the included studies. All six studies assessed muscle damage, with four evaluating CK and two assessing ALT. CK presented substantial post-race increases in every study, often reaching very high values, indicating marked acute muscle damage. ALT also rose consistently following competition, supporting the presence of exercise-induced muscular stress.

Regarding neuromuscular markers, four studies measured SJ and/or KE MVIC, all reporting post-race declines in performance, reflecting impaired force production immediately after the event.

For cardiac biomarkers, five studies analyzed CK-MB and/or cTnI, both of which showed transient post-race elevations suggestive of an acute and reversible cardiac stress response.

Due to methodological heterogeneity across studies, no meta-analysis was performed, and results were synthesized descriptively.

### 3.3. Reporting Bias

Reporting bias across studies was not formally assessed, as no quantitative synthesis was performed and the number of studies available for each outcome was limited.

## 4. Discussion

### 4.1. Muscle Damage Biomarkers

TR imposes physical demands that exceed those of other running modalities. The irregularity of the terrain requires constant activation of stabilizing musculature, while changes in slope involve continuous alternation between concentric and eccentric muscle actions [14]. Uphill sections substantially increase cardiovascular load and oxygen consumption, whereas downhill running predominantly triggers eccentric contractions that generate substantial mechanical stress and muscle damage [13], making this one of the most characteristic physiological consequences of TR.

During downhill segments, eccentric muscle activity is especially pronounced; muscles lengthen while controlling movement and must sustain loads greater than those encountered during concentric or isometric phases [59]. This downhill pattern, observed both in TR competitions and simulated protocols, induces significant increases in muscle damage biomarkers such as CK [37,49,50,58,60].

These findings are consistent across studies involving both amateur and high-level runners [50,61] linking prolonged eccentric loading during TR to structural muscle fiber damage [62,63]. Giovanelli et al. [41] and Saugy et al. [49] reported drastic increases in CK levels after TR events, in some cases exceeding 3000 U/L, indicating substantial but transient muscle damage.

However, variability in CK responses may be related to training status and prior physiological adaptations, which allow more highly trained individuals to tolerate greater workloads without clinical symptoms, as suggested by Pradas et al. [50].

ALT, another biomarker associated with muscle and hepatic stress, also increased significantly following TR in studies such as those by Lecina [37] and Pradas [50]. These elevations reinforce the concept that extreme effort induces a systemic inflammatory response, largely dependent on the magnitude of eccentric damage, which is influenced by slope, downhill duration, and the athlete’s level of adaptation.

### 4.2. Neuromuscular Function

Neuromuscular function refers to the capacity of the nervous system and muscles to work together efficiently during movement [17]. In the context of TR, this function is challenged by irregular terrain, continuous changes in running rhythm, and prolonged ascents and descents—particularly downhill, where neuromuscular performance can be markedly compromised. These alterations manifest as temporary impairments in force production [37,49,50,58]. The most commonly used assessments include SJ [37,50,58] and KE MVIC [14,49,58].

Studies on mountain runners show significant reductions in maximal voluntary force—particularly in the quadriceps—after events with a strong eccentric component, with some deficits persisting for several days [15,58]. All studies evaluating neuromuscular markers reported notable post-race declines in force production [14,37,49,50,58]. Coratella et al. [14] observed an average reduction of 18% in KE MVIC post-race, while Birat et al. [58] found that SJ values remained depressed even 48 h after competition, suggesting prolonged neuromuscular recovery. These findings align with those of Millet et al. [19], who previously demonstrated that eccentric load–induced neuromuscular fatigue in mountain running is greater than in flat running.

Beyond acute fatigue, repeated eccentric loading during TR induces micro-lesions in muscle tissue that contribute to both peripheral and central components of neuromuscular impairment. Importantly, available evidence indicates that these alterations are largely transient and tend to recover within days when adequate recovery is provided. Experimental and field studies in trail and endurance running suggest that, following recovery, runners may exhibit a repeated-bout effect, characterized by improved tolerance to eccentric loading and, in some cases, supercompensatory adaptations in strength and mechanical resilience [64,65]. These adaptive responses highlight the importance of appropriate recovery strategies to balance training stimulus and physiological restoration.

Neuromuscular fatigue affects not only force production but also motor control patterns, potentially increasing the risk of falls [7,8,66] and secondary injuries during competitions [63]. Neuromuscular changes observed in TR are directly linked to the degree of muscle damage, and recovery depends on the runner’s adaptation level and post-exercise management. Thus, a bidirectional relationship exists between muscle damage and neuromuscular function.

Despite these consistent findings, the studies reviewed [14,37,49,50,58] show considerable methodological heterogeneity—ranging from the type of neuromuscular test to the timing of post-race assessments—limiting comparability and preventing the establishment of standardized reference values.

### 4.3. Transient Cardiac Damage

Systemic alterations affecting various organs, including the heart, have been documented in TR. In this review, nine of the 15 included articles analyzed cardiac stress [41,44,45,53,54,55,57,58]. In long-duration events, some runners exhibited transient increases in cardiac biomarkers; however, no clear consensus exists regarding which markers are most effective for detecting cardiac stress [67,68]. This heterogeneity was reflected in the present review, where the following markers were assessed: CK-MB [41,45,53,56,58], hs-TnT [44,57], cTnI [41,54,55,56,58], and NT-proBNP [44,45,53,54,57,69]. Most studies assessing cTnI, CK-MB, hs-TnT, or NT-proBNP reported post-race increases, although not all markers were evaluated in every study [45,57].

These biomarkers have been interpreted as indicators of functional myocardial stress rather than permanent structural damage [20,70]. Although many biomarkers return to baseline within several days, some studies reported prolonged elevations—particularly ALT and NT-proBNP—depending on race duration and cumulative eccentric load [30,71].

This cardiac response likely represents part of the broader physiological adaptation to extreme exertion in TR, alongside muscle and renal stress episodes [72,73,74,75]. Similar findings have been reported in other endurance sports, such as triathlon and ultramarathon events, where cardiovascular stress induces troponin release without evidence of structural myocardial damage [68]. However, research specifically addressing cardiac stress in TR remains scarce, often relying on case studies or small sample sizes.

In addition to biochemical markers, several studies incorporated cardiac imaging to assess functional responses to ultra-endurance TR. The updated evidence shows minor but transient alterations in echocardiographic parameters after competition. Burger et al. [57] reported slight post-race reductions in LVEF and FAC, together with marked increases in left atrial volume index (LAVI), suggesting reversible cardiac fatigue and transient elevations in preload. Similarly, Vitiello et al. [55] observed marginal decreases in LVEF after an extreme ultra-trail event, while Klenk et al. [54] documented stable ventricular systolic function but dynamic changes in NT-proBNP and cTnI during a 4486 km multi-stage race. Collectively, these imaging findings reinforce the concept that long-duration TR induces acute, functional cardiac strain without evidence of persistent structural impairment.

Nonetheless, this review presents important limitations, including considerable heterogeneity in the performance level of participants, the absence of female representation (less than 10% of the total sample), and major methodological differences across the included studies (event type, biomarkers, measurement units, and participant level). These factors limit the comparability of results and the generalizability of findings. Additionally, commonly used physiological indicators in TR, such as heart rate responses during competition, could not be systematically analyzed due to their limited and inconsistent reporting in the available literature. Accordingly, no formal assessment of the certainty of evidence (e.g., GRADE) was performed.

## 5. Conclusions

TR produces substantial acute muscular, neuromuscular, and cardiac stress, particularly in events involving pronounced downhill sections and high eccentric loading. Across the included studies, consistent post-race increases were observed in CK and ALT, together with significant declines in neuromuscular performance indicators such as SJ and MVIC. These findings confirm that TR induces notable exercise-related muscle damage and an accompanying reduction in contractile efficiency.

Cardiac biomarkers such as CK-MB, cTnI, hs-TnT, and NT-proBNP also demonstrated transient post-exercise elevations, and the limited imaging data available showed minor but reversible alterations in parameters such as LVEF, FAC, and LAVI. Altogether, current evidence suggests an acute but reversible cardiac stress response rather than permanent myocardial injury, reflecting a temporary functional adaptation to the extreme physiological demands of TR.

However, the available literature shows considerable methodological heterogeneity, small sample sizes with poor female representation, which limits the generalizability of findings. More research is needed, especially involving women, different performance levels, and standardized protocols for assessing muscular and cardiac responses in TR.

Overall, this review highlights the importance of monitoring muscle and cardiac biomarkers, as well as neuromuscular function, to optimize training load, recovery strategies, and injury prevention in trail runners.

## Figures and Tables

**Table 1:** Characteristics of the runners and the events.

Author	Participants	Age ± SD (Years)	Level	Race	Distance (km)	Stages	Elevation Gain/Loss
Giovanelli et al. (2020) [41]	10	38.2 ± 12.4	NR	TR	22.48 and 40	3	NR
Lecina et al. (2022) [37]	4	38 ± 4.11	NR	U	768	11	46,865 m+
Saugy et al. (2013) [49]	9 8 CG	41.6 ± 13.1 29.3 ± 8.1	NR	U	330	1	24,000 m+
Coratella et al. [14]	10	22 ± 3	NR	T	30 (s: 10 km/h)	1	−20%
Birat et al. (2020) [58]	12	14.40	NR	U	48.2 and 66	2	NR
Pradas et al. (2021) [50]	20	43.3 ± 4.52	10 A 10 HT	U	108	1	5800 m+
Martinez-Navarro et al. (2019) [43]	46 M	42 ± 7.49	A	U	118	1	5439 m+ 4227 m−
Belli et al. (2018) [51]	6 M	47 ± 5	HT	U	217	1	12,200 m+ 12,200 m−
Rubio-Arias et al. (2019) [52]	6 M	30.5 ± 8	A	U	111	1	4474 m+ 4420 m−
Kim et al. (2012) [53]	20 EIH 10 CG	46.8 ± 1.23 47.5 ± 1.33	A	U	100	1	NR
Le Goff et al. (2022) [44]	17 M 11 F	M: 45.3 ± 7.8 F: 43.3 ± 9.8	A	U	105	1	5600 m±
Burger et al. (2024) [57]	14 M	42.9 ± 8.0	P	U	130	1	1170 m±
Klenk et al. (2021) [54]	18 F 2 M	47.8 ± 10.4	P	U	4486	64	NR
Vitiello et al. (2014) [55]	21 M	40 ±8	P	U	166	1	9600 m+
Wegberger et al. (2020) [56]	14 M 1 F	43 ± 9	HT	U	130	1	1170 m+

M: male; F: female; CG: control group; EIH: exercise-induced hypertension; SD: standard deviation; A: amateur; HT: highly trained; P: professional; TR: trail running; T: treadmill; U: ultra trail; s: speed; m: meters of elevation; km/h: kilometers per hour; m+: positive slope; m−: negative slope; m±: positive and negative slope; NR: not reported.

**Table 2:** Physical markers and biomarkers of muscular, neuromuscular, and cardiac damage.

Author	Muscular Damage		Neuromuscular Function		Cardiac Parameters	
Giovanelli et al. (2020) [41]	CK (U∙L^−1^) Pre: 211.7 ± 164.95 Post: 3637.6 ± 2754.8	ALT (U∙L^−1^) Pre: 21.6 ± 5.7 Post: 42.8 ± 14.74	NR		CK-MB (ng∙L^−1^) Pre: 4.42 ± 3.96 Post: 50.56 ± 35.99	cTnI (ng∙mL^−1^) Pre: 0.01 ± 0.0 Post: 0.02 ± 0.0
Lecina et al. (2022) [37]	CK (U∙L^−1^) Pre: 98.51 ± 24.53 Post: 974 ± 402.66 48 h: 474.85 ± 185.70 9 d: 88.0 ± 16.27	ALT (U∙L^−1^) Pre: 17.25 ± 3.59 Post: 44.75 ± 12.44 48 h: 316.00 ± 70.88 9 d: 208.00 ± 31.55	SJ (cm) Pre: 30.68 ± 2.46 Post: 22.05 ± 8.59		NR	
Saugy et al. (2013) [49]	G CK (U∙L^−1^) Pre: 112 ± 33 Post: 3719 ± 3045	CG CK (U∙L^−1^) Pre: 122.5 ± 41.1 Post: 147.7 ± 32.6	G KE MVIC (N) Pre: 390 Post: 290	CG KE MVIC (N) Pre: 370 Post: 320	NR	
Coratella et al. (2024) [14]	CK (pg∙mL^−1^) Pre: 32.92 ± 23,87 Post: 147.27 ± 55.44		KE MVIC (N) Pre: 608 ± 156 Post: 500 ± 109		NR	
Birat et al. (2020) [58]	NR		SJ (cm) Pre: 29.5 ± 4.7 Post: 26.7 ± 4.5	KE MVIC (N) Pre: 208.1 ± 49.2 Post: 194.9 ± 44.4	NR	cTnI (ng∙mL^−1^) Pre: 0.004 ± 0.005 Post: 0.143 ± 0.131 24 h: 0.033 ± 0.030 48 h: 0.013 ± 0.020
Pradas et al. (2021) [50]	A CK (U∙L^−1^) Pre: 164.3 ± 69.39 Post: 3251.6 ± 1011.89	HT CK (U∙L^−1^) Pre: 193.6 ± 42.11 Post: 4261.5 ± 1469.6	A SJ (cm) Pre: 25.5 ± 4.38 Post: 16.74 ± 3.91	HT SJ (cm) Pre: 27.12 ± 5.29 Post: 19.87 ± 4.31	NR	
Martinez-Navarro et al. (2019) [43]	NR	NR	NR	NR	NT-proBNP (ng∙L^−1^) Pre: 20.9 ± 15.84 Post: 443 ± 280.61	
Belli et al. (2018) [51]	CK (U∙L^−1^) Pre 132 ± 18 km 84: 3988 ± 1004 km 177: 18,667 ± 10,664 Post 19,157 ± 12,369	ALT (U∙L^−1^) Pre 28 ± 3 km 84: 109 ± 13 km 177: 677 ± 406 Post 668 ± 407	NR		NR	
Rubio-Arias et al. (2019) [52]	54 km *race* CK (U∙L^−1^) Pre: 886.6 ± 2187.9 Post: 2213.8 ± 2354.5 24 h: 1014.9 ± 732.6 48 h: 495.8 ± 358.0 72 h: 309.4 ± 191.6	111 km *race* CK (U∙L^−1^) Pre: 6174.0 ± 197.9 Post: 8976.0 ± 4327.1 24 h: 2132.5 ± 1399.6 48 h: 1277.7 ± 1368.2 72 h: 604.2 ± 878.3	NR		NR	
Kim et al. (2012) [53] †	EIH CK (U∙L^−1^) Pre: ~100 Post: ~3500	CG CK (U∙L^−1^) Pre: ~100 Post: ~1500	NR		EIH CK-MB (U∙L^−1^) Pre: ~10 Post: ~60 cTnI (ng∙L^−1^) Pre: ~0.02 Post: ~0.15 NT-proBNP (ng∙L^−1^) Pre: ~20 Post: ~320	CG CK-MB (U∙L^−1^) Pre: ~10 Post: ~30 cTnI (ng∙L^−1^) Pre: ~0.02 Post: ~0.24 NT-proBNP (ng∙L^−1^) Pre: ~20 Post: ~180
Le Goff et al. (2022) [44]	NR		NR		CK-MB (ng∙mL^−1^) Pre: 2.31 ± 1.25 Post: 91.9 ± 86.2 7 d: 5.15 ± 2.81	NT-proBNP (ng∙L^−1^) Pre: 41.7 ± 30.5 Post: 1190 ± 663 7 d: 29.5 ± 20.3
Burger et al. (2024) [57]	NR		NR		LVEF (%) Pre: 57.0 ± 6.7 Post: 55.4 ± 5.7 GLS (%) Pre: 19.0 ± 1.8 Post: 19.2 ± 1.9 GFWS (%) Pre: 26.4 ± 2.4 Post: 24.8 ± 2.7	LAVI (mL∙m^−2^) Pre: 28.4 ± 7.3 Post: 48.0 ± 4.6 FAC (%) Pre: 48.0 ± 4.6 Post: 46.7 ± 3.8
Klenk et al. (2021) [54]	NR		NR		cTnI (ng∙mL^−1^) Pre: 7 ± 3 km 1735: 17 ± 10 km 3369: 17 ± 10 LVEF (%) Pre: 64 ± 4 km 1735: 65 ± 5 km 3369: 65 ± 6	NT-proBNP (ng∙L^−1^) Pre: 30 ± 23 km 1735: 136 ± 178 km 3369: 111 ± 87 RVEF (%) Pre: 57 ± 4 km 1735: 57 ± 5 km 3369: 59 ± 6
Vitiello et al. (2014) [55]	NR		NR		cTnI (ng∙L^−1^) Pre: 0.010 ± 0.001 Post: 0.038 ± 0.055	LVEF (%) Pre 53.4 ± 4.9 Post 52.0 ± 4.3
Wegberger et al. (2020) [56]	CK (U∙L^−1^) Pre: NR Post: 6992		NR		cTnI (ng∙L^−1^) Pre: NR Post: 0.056	NT-proBNP (ng∙L^−1^) Pre: NR Post: 723

† Values for Kim et al. [53] were approximately extracted from published figures, as numerical data were not provided in the original article. CK: creatine kinase; N: newtons; ALT: alanine aminotransferase; KE MVIC: knee extensor maximal voluntary isometric contraction; SJ: squat jump; CK-MB: creatine kinase isoenzyme MB; cTnI: cardiac troponin I; NT-proBNP: N-terminal pro–B-type natriuretic peptide; hs-TnT: high-sensitivity cardiac troponin T; FAC: fractional area changes; GFWS: global free wall strain; GLS: global longitudinal strain; LAVI: left atrial volume index; LVEF: left ventricular ejection fraction; RVEF: right ventricular ejection fraction; A: amateur; HT: highly trained; P: professional; G: group; CG: control group; h: hours; d: days; EIH: exercise-induced hypertension group.

## Data Availability

Further inquiries can be directed to the corresponding author.

## Supplementary Materials

The following supporting information can be downloaded at: https://www.mdpi.com/article/10.3390/muscles5010009/s1, Table S1. Full electronic search strategy for PubMed (last searched: 31 August 2025). Supplementary File S1: PRISMA 2020 Checklist for Systematic Reviews. Supplementary File S2: PRISMA 2020 Checklist for Abstracts [76].

## Abbreviations

The following abbreviations are used in this manuscript:

TR	Trail Running
CK	Creatine Kinase
LDH	Lactate Dehydrogenase
ALT	Alanine Aminotransferase
MB	Myoglobin
LVEF	Left Ventricular Ejection Fraction
HRV	Heart Rate Variability
cTn	Cardiac Troponin
hs-cTn	High-sensitivity Troponin
CK-MB	Creatine Kinase MB isoenzyme
NT-proBNP	N-Terminal pro–B-type Natriuretic Peptide
PRISMA	Preferred Reporting Items for Systematic Reviews and Meta-Analyses
NIHR	National Institute for Health and Care Research
PROSPERO	International Prospective Register of Systematic Reviews
MeSH	Medical Subject Headings
AST	Aspartato Aminotransferasa
SJ	Squat Jump
CMJ	Counter-Movement Jump
ABA	Abalakov Jump
MVIC	Maximal Voluntary Isometric Contractions
Pro-BNP	Pro–B-type Natriuretic Peptide
cTnI	Cardiac Troponin I
hs-cTnI	High-sensitivity cardiac Troponin I
NT-proBNP	N-terminal pro–Brain Natriuretic Peptide
PEDro	Physiotherapy Evidence Database
NHLBI	National Heart, Lung, and Blood Institute
hs-TnT	Troponin T High Sensitivity
FAC	Fractional Area Changes
LAVI	Left Atrial Volume Index
M	Male
F	Female
CG	Control Group
SD	Standard Deviation
A	Amateur
HT	Highly Trained
P	Professional
T	Treadmill
U	Ultra Trail
S	Speed
m	Meters of elevation
km/h	Kilometers per hour
NR	Not Reported
N	Newtons
GFWS	Global Free Wall Strain
GLS	Global Longitudinal Strain
G	Group
h	Hours
d	Days
