# Supplementary material for: Muscle, Neuromuscular, and Cardiac Damage in Trail Running: A Systematic Review
Source: Muscles. 2026 Jan 29;5(1):9. doi: 10.3390/muscles5010009 (PMC12921809; doi:10.3390/muscles5010009)
Supplement: Supplementary file 1 [file muscles-05-00009-s001.zip › Supplementary Table S1.pdf]

Table S1. Full electronic search strategy for PubMed (last searched: 31 August 2025).

Database	Platform	Search date	Full search strategy (copy/paste)	Limits / filters
PubMed	NCBI	31 Aug 2025	(("trail running"[Title/Abstract] OR "trail run*"[Title/Abstract] OR "mountain running"[Title/Abstract] OR "ultra-trail"[Title/Abstract] OR ultratrail[Title/Abstract] OR "ultra trail"[Title/Abstract]) AND (("muscle damage"[Title/Abstract] OR "muscle injur*"[Title/Abstract] OR "exercise-induced muscle damage"[Title/Abstract] OR "Creatine Kinase"[MeSH Terms] OR "creatine kinase"[Title/Abstract] OR CK[Title/Abstract] OR "Lactate Dehydrogenase"[MeSH Terms] OR "lactate dehydrogenase"[Title/Abstract] OR LDH[Title/Abstract] OR myoglobin[Title/Abstract] OR "Alanine Transaminase"[MeSH Terms] OR "alanine aminotransferase"[Title/Abstract] OR ALT[Title/Abstract]) OR ("neuromuscular fatigue"[Title/Abstract] OR "neuromuscular function"[Title/Abstract] OR "Neuromuscular Junction"[MeSH Terms] OR "Muscle Fatigue"[MeSH Terms] OR "maximal voluntary isometric contraction"[Title/Abstract] OR MVIC[Title/Abstract] OR "squat jump"[Title/Abstract] OR SJ[Title/Abstract]) OR ("cardiac biomarker*"[Title/Abstract] OR troponin[Title/Abstract] OR "Troponin I"[MeSH Terms] OR "Creatine Kinase, MB Form"[MeSH Terms] OR "CK-MB"[Title/Abstract] OR "Natriuretic Peptide, Brain"[MeSH Terms] OR NT-proBNP[Title/Abstract] OR BNP[Title/Abstract] OR "high-sensitivity cardiac troponin"[Title/Abstract] OR hs-cTnI[Title/Abstract] OR hs-cTnT[Title/Abstract])) AND (prevention[Title/Abstract] OR performance[Title/Abstract] OR recovery[Title/Abstract])) AND ("2010/01/01"[Date - Publication] : "2025/08/31"[Date - Publication]) AND (english[Language] OR spanish[Language]) AND Humans[MeSH Terms]	Date of publication: 01 Jan 2010–31 Aug 2025; Languages: English or Spanish; Species: Humans
